# Effect of Propolis Nanoparticles against *Enterococcus faecalis* Biofilm in the Root Canal

**DOI:** 10.3390/molecules26030715

**Published:** 2021-01-30

**Authors:** Abhishek Parolia, Haresh Kumar, Srinivasan Ramamurthy, Thiagarajan Madheswaran, Fabian Davamani, Malikarjuna Rao Pichika, Kit-Kay Mak, Amr S Fawzy, Umer Daood, Allan Pau

**Affiliations:** 1 Division of Clinical Dentistry, School of Dentistry, International Medical University, Kuala Lumpur 57000, Malaysia; umerdaood@imu.edu.my; 2 School of Medicine, International Medical University, Kuala Lumpur 57000, Malaysia; HareshKantilal@imu.edu.my; 3 College of Pharmacy & Health Sciences, University of Science and Technology of Fujairah, Fujairah, United Arab Emirates; s.ramamurthy@ustf.ac.ae or; 4 Department of Pharmaceutical Technology, International Medical University, Kuala Lumpur 57000, Malaysia; thiagarajan@imu.edu.my; 5 School of Health Sciences, International Medical University, Kuala Lumpur 57000, Malaysia; fabian_davamani@imu.edu.my; 6 Department of Pharmaceutical Chemistry, School of Pharmacy, International Medical University, Kuala Lumpur 57000, Malaysia; mallikarjunarao_pichika@imu.edu.my (M.R.P.); kitkaymak@studentimuedu.onmicrosoft.com (K.-K.M.); 7 UWA Dental School, University of Western Australia, Nedlands, WA 6009, Australia; amr.fawzy@uwa.edu.au; 8 School of Dentistry, International Medical University, Kuala Lumpur 57000, Malaysia; allan_pau@imu.edu.my

**Keywords:** dentinal tubule disinfection, *Enterococcus faecalis*, propolis nanoparticle

## Abstract

To determine the antibacterial effect of propolis nanoparticles (PNs) as an endodontic irrigant against *Enterococcus faecalis* biofilm inside the endodontic root canal system. Two-hundred-ten extracted human teeth were sectioned to obtain 6 mm of the middle third of the root. The root canal was enlarged to an internal diameter of 0.9 mm. The specimens were inoculated with *E. faecalis* for 21 days. Following this, specimens were randomly divided into seven groups, with 30 dentinal blocks in each group including: group I—saline; group II—propolis 100 µg/mL; group III—propolis 300 µg/mL; group IV—propolis nanoparticle 100 µg/mL; group V—propolis nanoparticle 300µg/mL; group VI—6% sodium hypochlorite; group VII—2% chlorhexidine. Dentin shavings were collected at 200 and 400 μm depths, and total numbers of CFUs were determined at the end of one, five, and ten minutes. The non-parametric Kruskal–Wallis and Mann–Whitney tests were used to compare the differences in reduction in CFUs between all groups, and probability values of *p* < 0.05 were set as the reference for statistically significant results. The antibacterial effect of PNs as an endodontic irrigant was also assessed against *E. faecalis* isolates from patients with failed root canal treatment. Scanning electron microscopy (SEM) and confocal laser scanning microscopy (CLSM) were also performed after exposure to PNs. A Raman spectroscope, equipped with a Leica microscope and lenses with curve-fitting Raman software, was used for analysis. The molecular interactions between bioactive compounds of propolis (Pinocembrin, Kaempferol, and Quercetin) and the proteins Sortase A and β-galactosidase were also understood by computational molecular docking studies. PN300 was significantly more effective in reducing CFUs compared to all other groups (*p* < 0.05) except 6% NaOCl and 2% CHX (*p* > 0.05) at all time intervals and both depths. At five minutes, 6% NaOCl and 2% CHX were the most effective in reducing CFUs (*p* < 0.05). However, no significant difference was found between PN300, 6% NaOCl, and 2% CHX at 10 min (*p* > 0.05). SEM images also showed the maximum reduction in *E. faecalis* with PN300, 6% NaOCl, and 2% CHX at five and ten minutes. CLSM images showed the number of dead cells in dentin were highest with PN300 compared to PN100 and saline. There was a reduction in the 484 cm^−1^ band and an increase in the 870 cm^−1^ band in the PN300 group. The detailed observations of the docking poses of bioactive compounds and their interactions with key residues of the binding site in all the three docking protocols revealed that the interactions were consistent with reasonable docking and IFD docking scores. PN300 was equally as effective as 6% NaOCl and 2% CHX in reducing the *E. faecalis* biofilms.

## 1. Introduction

The main objective of endodontic therapy is to eradicate microbial infections from the involved root canal system. [1,2]. Microorganisms in the root canal space can attach to each other and grow into a biofilm as a microbial community on the dentin walls and pose challenges for disinfection [3,4]. Root canal disinfection can be facilitated by mechanical and chemical means via irrigation with endodontic irrigants [5,6,7]. However, bacteria in mature biofilm can resist the action of antibacterial irrigants and are remarkably difficult to eradicate [8]. Within a biofilm, a wide variety of bacteria are found forming a multi-species community; however, *Enterococcus faecalis* is one of the commonly isolated species that may play a role in persistent endodontic infections [9,10,11] due to inherent antimicrobial resistance, ability to adapt to harsh environmental changes, and the ability to invade into the dentinal tubules. They are protected from endodontic irrigants and are therefore difficult to eliminate [12,13]. Several studies have evaluated the antimicrobial efficacy of endodontic irrigants such as sodium hypochlorite (NaOCl) [14], ethylene diamine tetra acetic acid (EDTA) [15], chlorhexidine (CHX) [16], and MTAD [17] against *E. faecalis* biofilms [18,19]. In general, the aim of any disinfection strategy is to reduce the bacterial load to a subcritical level so that the patient’s immune response allows healing by itself [12]. Endodontic research has always been focused on developing methods/endodontic irrigants that can completely remove the bacterial biofilm with minimum side-effects.

Several endodontic irrigants are being widely used in the treatment of biofilms with varied effectiveness. Although 2% CHX has been proven to possess a high antimicrobial property, it has failed to disrupt the biofilm. However, NaOCl can disrupt the biofilm, but it is a known irritant to periapical tissues. Therefore, identification of natural products in the disinfection of root canals can be interesting. Propolis, a natural product, has also been attempted as an endodontic irrigant in the recent past, has shown to be effective against *E. faecalis* [20,21], and its antibacterial effect has been attributed to its chemical composition [22]. Raw propolis is composed of aromatic oils and plant resins depending on its botanical origin and harvesting seasons [23]. Due to its high diversity in chemical composition, propolis possesses a large variation in biological and pharmaceutical properties [24]. Additionally, there has been increasing interest in using nanoparticles in clinical endodontics due to its enhanced drug stability, treatment efficacy, and penetration power compared to a pure drug solution [25,26,27]. Novel packages based on nanomaterials can be active, bioactive, or intelligent. Nanoparticles with their unique physicochemical properties, such as ultrasmall sizes, large surface area/mass ratio, and increased chemical reactivity, have led research toward new prospects of treating and preventing dental infections [25]. The capacity of nano-antimicrobial agents to penetrate bacteria and the biofilm backbones can make them potential agents for controlling increasing infections [28]. Surface adhesins derived from bacteria are anchored on the bacterial cell surface via sortase A [29], a transpeptidase that covalently links the LPXTGX motif-containing surface proteins to the cell wall of Gram-positive bacteria [30]. Sortase A plays a critical role in modulating the surface properties of bacteria that adhere to the tooth surface and contributes to cariogenicity [31]. Therefore, the aim of the present study was to evaluate the antibacterial effect of propolis nanoparticles (PNs) as an endodontic irrigant against *E. faecalis* biofilm in root canal dentinal tubules at depths of 200 and 400 μm and compare it with different endodontic irrigants. Antibacterial analysis and propolis-induced membrane protein damage in bacteria were also understood at the molecular level using computational molecular simulations, which has not been performed in previous studies. The null hypothesis tested was that PN300, PN100, saline, P300, P100, 6% NaOCl, and 2% CHX as irrigants do not show any difference in reducing *E. faecalis* CFUs in the root canal dentinal tubules.

## 2. Materials and Methods

The antibacterial effectiveness of PN as an endodontic irrigant was evaluated against the strain *E. faecalis* (ATCC 29212) in a human tooth model. The study was approved by IMU Joint Committee on Research and Ethics under the research project ERGS/1/2013/SKK11/IMU/03/01. Two hundred and ten extracted human anterior teeth (21–35 years) were used in this study and ethical approval was received from the Institutional Review Board at International Medical University.

### 2.1. Preparation of Ethanolic Extracts of Malaysian Propolis

Malaysian propolis was collected from a bee farm, Pahang, Malaysia with the geographical coordinates: north latitude 3.8126°, east latitude 103.3256°, and height of 12 m above sea level. The extraction method used in this study was similar to the method explained by Jacob et al. [32]. To study the content of Malaysian propolis, reversed phase high performance liquid chromatography (RP-HPLC) analysis was carried out. The flavonoids such as pinocembrin (5.90 µg/mL), kaempferol (5.88 µg/mL), and quercetin (1.43 µg/mL) were identified to be in the highest concentration in Malaysian propolis [33].

### 2.2. Preparation and Characterization of PN

**Figure 1:**
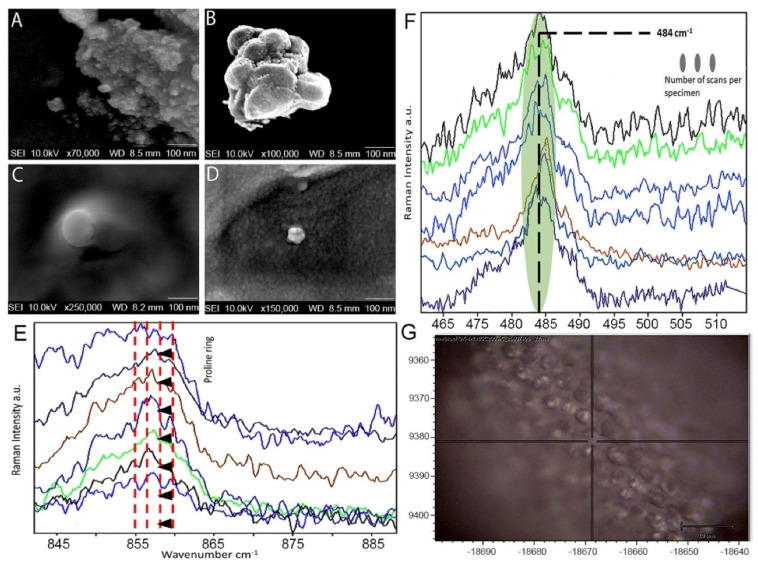
(**A**–**D**) The morphology of the developed propolis nanoparticles investigated with the aid of SEM, showing spherical shapes and agglomerations. (**E**) Representative Raman spectra recorded in the region of different experimental specimens where there is a proline bond at 855 cm^−1^ and (**F**) 484 cm^−1^ regions; (**G**) a representative spectral region drawn for Raman peaks.

Propolis nanoparticles were prepared by the ultrasonication method: 0.01 g Propolis and 0.1 g Tween 80 (stabilizer) were added to conical centrifuge tubes (Life Sciences, IN, USA) containing 9.89 g distilled water. The mixture was mixed in a vortex mixer (Stuart Model SA8, Bibby Scientific, UK) for 1 min followed by sonication for 20 min using a probe-type sonicator. To avoid thermal degradation of the propolis during sonication and formulation, tubes were kept in an ice bath. The particle size distribution and polydispersity index of the propolis nanoparticles were determined by dynamic light scattering using a Zeta-sizer Nano S90 (Malvern, Worcestershire, UK) at a fixed angle of 90° with a helium-neon laser, 4 mW operating at 633 nm. The formulation was suitably diluted with distilled water and measured at 25 °C. Data were collected after 2 min of equilibration time and averaged over three measurements (Figure 1).

### 2.3. Antibacterial Effect of PN as an Endodontic Irrigant Against E. faecalis (ATCC 29212) in Human Tooth Model

#### 2.3.1. Dentine Block Specimens

The experiments were carried out on an extracted human tooth model similar to the study conducted by Chua et al. [34]. A total of 210 sound human anterior teeth including maxillary anterior teeth and mandibular canines with complete root formation were included in this study. Teeth with root caries and resorption were excluded from the study. The teeth were cleaned and stored in saline during all procedures to avoid dehydration. A low-speed diamond disc (Bredent^®^, Wittighausen, Senden, Germany) mounted on a milling machine (10,000 rpm) under water cooling was used to section the teeth between the cementoenamel junction and the apical third of the root. Approximately 6 mm of the middle third of the roots was obtained. Peeso Reamer no. 2 (Mani^®^, Utsunoniya, Tochigi, Japan) in a low-speed hand piece (Kavo, Charlotte, NC, USA) was used to standardize the internal diameter of root canals to 0.9 mm. Dentine blocks were subjected to sonic irrigation (Endoactivator, Dentsply, Weybridge, Surrey, UK) using 5.25% NaOCl (Clorox^®^, Oakland, CA, USA) and then 17% EDTA (Calasept^®^, Nordiska Dental, Ängelholm, Skåne Country, Sweden) for two minutes to remove the smear layer. The action of hypochlorite was neutralized by using 5% sodium thiosulfate for ten minutes. Thereafter, the dentine block specimens were thoroughly rinsed with sterile saline after each irrigation. Following this, the dentine blocks were sterilized by autoclave (LTE^®^, Oldham, Lancashire, UK) at 121 °C for 20 min. In order to prevent any contact of *E. faecalis* and medicament with the external surface, nail varnish was applied to the outer surface of the specimen. Petri dishes containing wax with a flat surface were prepared, and the surface was disinfected using 70% ethanol, later air dried in a sterile biosafety cabinet. All experiments were performed in a laminar hood after ultraviolet sterilization. Dentine block specimens were placed upright with the apical ends fixed to the petri dishes with wax (Premiere Dental, Malaysia), using a thin small square of sterilized parafilm (Parafilm M^®^, Brand, Wertheim, Baden-Württemberg, Germany) obliterating the apical orifice to prevent any softened wax from entering the root canals.

#### 2.3.2. *E. faecalis* Inoculation and Biofilm Formation

*E. faecalis* were suspended in 20.0 mL of tryptic soy broth (TSB) (BD DifcoTM, NJ, USA). The cell suspension was adjusted to match the turbidity of 1.5 × 10^8^ CFUs/mL (equivalent to 0.5 McFarland standards). The *E. faecalis* inocula were transferred into the dentine block specimens using sterile 5.0 mL syringes (Terumo^®^, Somerset, NJ, USA) with 30-gauge needles (Terumo, Somerset, NJ, USA) in a sterile laminar flow hood. The coronal part of the dentine blocks was then sealed immediately using parafilm (Parafilm M^®^, Brand, Wertheim, Baden-Württemberg, Germany). Following this inoculation, dentine block specimens were incubated for 21 days at 37 °C. The root canals were replenished with *E. faecalis* inoculum every three days to supply nutrients to the bacteria and prevent their death.

#### 2.3.3. Endodontic Irrigant Placement

Following the inoculation period, 210 dentine blocks were randomly divided into seven groups (n = 30) according to different endodontic irrigant treatments: Group I—Saline; Group II—Propolis 100 µg/mL (P100); Group III—Propolis 300 µg/mL (P300); Group IV—Propolis Nanoparticles 100 µg/mL (PN100); Group V—Propolis Nanoparticles 300 µg/mL (PN300); Group VI—6% Sodium Hypochlorite (6%NaOCl) (Calasept; Upplands Väsby; Sweden); Group VII—2% Chlorhexidine (2%CHX) (Calasept, Upplands Väsby, Sweden). Five milliliters of each endodontic irrigant was placed into the root canal using a 30-gauge side vented needle (Endo-EZE, Ultradent, South Jordan, UT, USA). Each group was further divided into three subgroups based on the time period for 1, 5, and 10 min.

#### 2.3.4. Raman Data Acquisition

All biofilms on dentin blocks (n = 5) treated with different irrigants were subjected to Raman spectroscopy. Designated dentin blocks were removed from culture plates and dried for 30 min at 35 °C. The blocks were placed on the XYZ axis positioning stage and areas of 15 μm were subjected to Raman analysis. The following parameters were employed for spectrum acquisition using a 100× objective: 514.5 nm green laser excitation, 785 nm with argon ion (spectral resolution 1.6 cm^−1^). Fifteen frames of 20 s each were recorded, and spectra were normalized to 1454 cm^−1^ (CH2 deformation peaks using Origin Pro. 8.5.1. software (Origin Lab Corp., Northampton, MA, USA).

#### 2.3.5. Dentinal Shavings Collection and Assessment

At the end of the experimental periods, the dentin blocks were removed from the petri dishes and the canals were dried with sterile paper points. Samples of dentinal shavings were collected after one minute, five minutes, and ten minutes of exposure. Dentinal shavings were collected using a Peeso reamer (Mani^®^, Utsunoniya, Tochigi, Japan) size no. 4 equivalent to 1.3 mm diameter, followed by size no. 6 equivalent to 1.7 mm diameter using a low-speed (1000 rpm) handpiece (Kavo^®^, Charlotte, NC, USA). Only one stroke was made to standardize the volume of dentinal debris collected.

The collected dentinal shavings were transferred into a micro-centrifuge tube (Axygen, NY, USA) containing 1 mL sterile tryptic soy broth (TSB) (BD DifcoTM, NJ, USA). A sterile micro tip was used to take 0.1 mL of broth containing dentinal shavings and transfer it to another tube containing 0.9 mL sterile TSB (BD DifcoTM, NJ, USA) broth. The content of each tube was serially diluted from 10^−1^ until 10^−4^. Three hundred microliters of the diluted shavings was spread evenly using an L-shaped glass rod. These TSB agar plates (BD DifcoTM, NJ, USA) were incubated for 24 h at 37 °C and colonies were counted with a tabulation of readings.

### 2.4. Scanning Electron Microscopy Characterization

HR-FESEM (Carl Zeiss Microscopy GmbH, Jena, Germany) was used for particle characterization. A small portion of the sample was coated with an ultra-thin gold layer (5 nm) for 30 s by a sputtering machine (Pumped Sputter Coater/Carbon Coater; Quorum Technologies Ltd., East Sussex, UK). The images were taken under high vacuum at 25 keV accelerating voltage.

Dentinal blocks (n = 3) were prepared using the same method as mentioned above under the dentine block specimens. A low-speed diamond disc (Bredent^®^, Wittighausen, Senden, Germany) mounted on a milling machine under water cooling was used to section the teeth between the cementoenamel junction and the apical third of the root to obtain 6 mm of the middle third of the roots. Peeso Reamer no. 2 (Mani^®^, Utsunoniya, Tochigi, Japan) in a low-speed hand piece (Kavo, Charlotte, NC, USA) was used to standardize the internal diameter of root canals to 0.9 mm. *E. faecalis* (ATCC 29212) was cultured in 10 mL TSB added with 8% sucrose with pH 7.4 and a minimal amount of xylitol (0–2%) at 37 °C for 48 h. This broth was incubated at 37 °C for 24 h. After centrifugation using 4000 rpm for 15 min, each cell pellet was washed thrice with sterile phosphate buffered solution (0.01 M, pH 7.2). Thereafter, it was re-suspended in 10 mL of the growth medium to adjust its concentration similar to 0.5 McFarland units (10^8^ cells/mL) before use. The bacterial inoculum was mixed in five milliliters of TSB and transferred into the root canal using sterilized syringes for a period of 21 days. After 21 days, intracanal irrigants were placed according to the groups mentioned above. Two parallel grooves were created using a diamond disc onto the external surfaces of the dentin specimen in mesiodistal direction to facilitate a split fracture. Final splitting was performed using a chisel and hammer. Following this, all specimens were dehydrated in ascending grades of ethanol for 20 min each and immediately transferred into the pressure chamber of the critical point drying machine (CPD 30; Leica). All specimens were mounted on aluminum stubs using double-sided conductive tape and 30 nm-thick layer gold sputtering was performed for two minutes. Following this, the specimens were examined using SEM (Philips/FEI XL30 FEG SEM) at an accelerated voltage of 5 kV at different magnifications and images were evaluated. Different magnifications and images were observed to evaluate the qualitative reduction in *E. faecalis*. A four-score scale system based on the percentage of residual isolated microbial cells was used to assess the microbial coverage on SEM images of the canal walls [35]. The scores were defined as clean dentin or residual isolated microbial cells covering less than 5% of the dentine, covering 5%–33% of the dentine, 34%–66% of dentine, and 67%–100% of the dentine.

### 2.5. Confocal Laser Scanning Microscopy Analysis

Confocal analysis was conducted to evaluate the effectiveness of PN300 and PN100 as endodontic irrigants. This was performed by assessing the viability profile. The percentage of live and dead bacteria was determined by fluorescent staining followed by imaging [36]. After the disinfection solution regimen, the specimen (n = 7 in each group) was rinsed in 0.1% by weight fluorescein for 24 h. Specimens were thereafter rinsed with deionized water and examined using CLSM (Leica Fluoview FV 1000, Olympus, Tokyo, Japan) equipped with a 60×/1.4 NA oil immersion lens using 488 nm argon/helium and a 633 nm krypton ion laser illumination in reflection as well as fluorescence modes. Reflected and fluorescence signals were detected using a photomultiplier tube to a depth of 20 μm and then converted to single-projection images for better visualization and qualitative analysis. Stacks of fluorescent images of the biofilm were obtained and examined using BioimageL software (v.2.0. Malmő, Sweden). This software provides information on the structure of the biofilm, including green staining to indicate live bacteria, red staining to indicate dead bacteria, and volume on a two-dimensional x–y section based on color segmentation algorithms written in MATLAB. The respective percentages for live and dead bacteria for each group were calculated.

### 2.6. Antibacterial Effect of PN as an Endodontic Irrigant Against E. faecalis Isolates from Patients with Failed Root Canal Treatment

#### 2.6.1. Patient Selection

Ten patients aged between 20 and 60 years were selected from those who attended the Oral Health Centre, Kuala Lumpur, Malaysia, needing endodontic retreatment. A detailed medical and dental history was obtained from each patient. Patients who have systemic disease or have received antibiotic treatment during the last three months were excluded from the study to minimize any risk of bias. Ten teeth from ten different patients with failed root canal treatment were included in this experiment. The main reasons observed were underextended root canal fillings, coronal leakage, and the presence of voids in the root canal fillings. Failure of root canal treatment was determined based on clinical examination such as the presence of pain, tenderness, swelling, sinus opening and mobility, and radiographical examinations such as persistence of periapical lesion and root resorption.

#### 2.6.2. Sampling Procedure

After explaining the complete process of investigation including the method of sample collection, a written informed consent was obtained. Thereafter, the retreatment procedure was carried out. An access cavity was prepared under syringe irrigation using sterile high-speed diamond bur under the rubber dam isolation. Root-filling material was removed by rotary instrumentation and K-files (Dentsply-Maillefer, Ballaigues, Switzerland) in a crown-down technique without the use of chemical solvent, accomplished by irrigation with sterile saline. Following this, a sterile paper point (Dentsply-Maillefer, Ballaigues, Switzerland) was then introduced into the full length of the canal and retained in position for one minute for sampling. The culture procedure was performed using the selective *E. faecalis* plates (Slanetz Bartley Agar (m-Enterococcus A.), Liofilchem, Italy) and the CFUs performed. Thereafter, the grown *E. faecalis* were suspended in 20.0 mL of TSB. The cell suspension was adjusted to match the turbidity of 1.5 × 10^8^ CFUs/mL (equivalent to 0.5 McFarland standards). One milliliter of *E. faecalis* suspension was transferred into an Eppendorf tube in addition to 50 microliters of each irrigant according to these seven groups including: group I—saline; group II—P100; group III—P300; group IV—PN100; group V—PN300; group VI—6% NaOCl; group VII—2% CHX. Fifty microliters of each endodontic irrigant was placed into 1 mL of *E. faecalis* suspension in an Eppendorf tube for one, five, and ten minutes. After this exposure, the content of each tube was serially diluted, and this was spread evenly onto the agar plate using an L-shaped glass rod and triplicated on three occasions. These plates were incubated for 24 h at 37 °C, the colonies were grown, and the microbial colony-forming units (CFU/mL) were counted.

### 2.7. Molecular Simulation

The present study was undertaken to determine the molecular interactions between bioactive compounds of propolis (Pinocembrin, Kaempferol, and Quercetin) and the proteins Sortase A and β-galactosidase by computational molecular docking studies. Molecular docking studies were carried out on crystal structures of sortase A, PDB ID: 4TQX, and β galactosidase, PDB ID: 6TBI. The glide (Standard precision (SP) mode and Extra precision (XP) mode) and induced-fit docking modules of Schrodinger 2020-2 were used to determine the interactions between bioactive compounds and proteins. From the binding energy and interaction studies, all three bioactive compounds have shown favorable interactions with amino acids in the active sites of Sortase A and β-galactosidase.

#### 2.7.1. Protein Preparation

The crystal structures of sortase A (PDB ID: 4TQX, X-ray resolution: 1.37 A°) and β-galactosidase (PDB ID: 6TBI, X-ray resolution: 1.46 A°) were downloaded from https://www.rcsb.org/. The proteins were prepared for molecular docking studies using protein preparation wizard with an OPLS-3e force field at pH 7.20 ± 0.20 and the other default settings.

#### 2.7.2. Active Site Grid Generation

The active site in sortase A (PDB ID: 4TQX) was defined using the amino acid residue, cys 205 as the centroid of the cavity, and the grid around the active site was generated using the receptor grid generation module with OPLS-3e force field and default settings. The active site in β-galactosidase (PDB ID: 6TBI) was defined based on the position of the inbound ligand (N8V) and the grid around the active site was generated using the receptor grid generation module with the OPLS-3e force field and default settings.

#### 2.7.3. Molecular Docking Studies

The glide module was used to dock the bioactive compounds into the active site grid using the standard precision (SP) and extra precision (XP) docking protocols with default settings. In the SP and XP docking protocols, the ligand was made flexible and the receptor was made rigid. To further confirm the binding efficacy of the bioactive compounds, induced-fit docking studies were performed using the “Induced-Fit docking” module. In this, the ligand was made flexible and all the residues within the range of 5 Å of the receptor were made flexible. In general, induced-fit docking provides better insights on binding interactions and efficacy. The poses with the highest negative docking scores are shown in the results.

Total numbers of colony forming units were calculated for assessing the remaining vital viable microbial population. SPSS computer software version 21.0 (SPSS Inc., Chicago, IL, USA) was used to perform the statistical analysis. The values were analyzed using the non-parametric Kruskal–Wallis and Mann–Whitney U tests to compare the reduction in *E. faecalis* between all intracanal irrigants. Probability values of *p* < 0.05 were set as the reference for statistically significant results.

## 3. Results

The accuracy of the methodology was validated by observing the large amount of *E. faecalis* CFUs in the saline (control group) at all experimental timings. Propolis nanoparticles sizes were observed in the range of 117.6 ± 5.6 nm with a polydispersity index (PDI) of 0.277 ± 0.011. Polydispersity index is an indicator of the size distribution of nanoparticles. The polydispersity index of PNs in this study was found to be 0.277 ± 0.011, signifying a low size profile and homogenous distribution. A polydispersity index that is equal to 1 signifies a solution having a broad and variable nanoparticle size distribution.

The morphology of the developed propolis nanoparticles was investigated with the aid of SEM, as illustrated in Figure 1. As illustrated in Figure 1A–D, the nanoparticles demonstrated spherical shape and agglomerations with an average size of about 150 nm, which is in agreement and close to the size values obtained from the polydispersity index, as the particles showed a smooth structure. Figure 1 provides comparative Raman spectra of all specimens, showing bands responsive to the treatment provided typifying changes within the absorbance spectrum. Significant and obvious changes with variations were seen at the 484 cm^−1^ regions according to the compounds used. These bands are signature peaks for the glycosidic link or the ring wagging for carbohydrates and polysaccharides due to linkages. There was a striking difference observed in specimens treated with PN300 as the bands reached the lowest intensity. This is suggestive of a decrease in the carbohydrate content within the biofilm, a factor more pronounced with the propolis nanoformulation and propolis itself. The specimens treated with saline showed the highest peaks, suggestive of C-H deformations coupled with C-O and C-C stretches and complexes. The deformation changes around the 855–857 cm^−1^ region are for amino acids and proline representing functional groups, with the highest peaks found for the PN300 groups.

**Figure 2:**
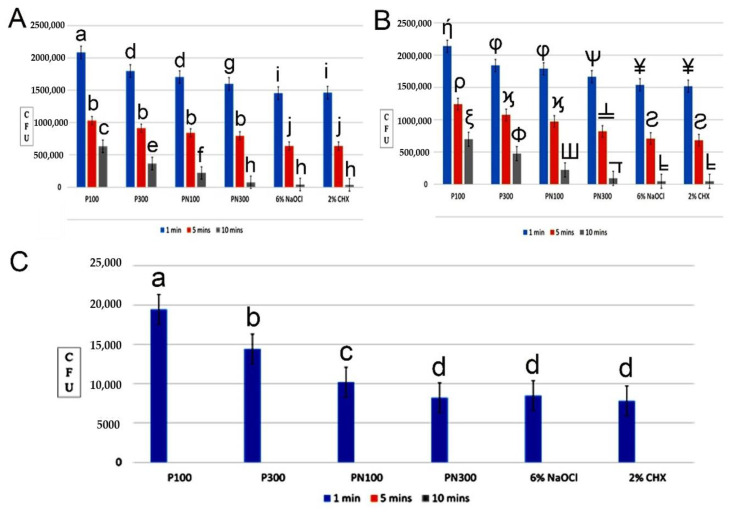
(**A**) Mean reduction in CFU at 1, 5, and 10 min at 200 µm dentinal tubule depth. (**B**) Mean reduction in CFU at 1, 5, and 10 min at 400 µm dentinal tubule depth. (**C**) Comparison of CFUs count between propolis nanoparticles (PNs) and other endodontic irrigants at 1, 5, and 10 min against *Enterococcus faecalis* isolates from patients with failed root canal treatment. Groups identified by different alphabets and symbols were significantly different at *p* < 0.05 in columns, respectively. The alphabets and symbols are in quasi-alphabetical order and are ordered according to the statistical difference between groups.

All the endodontic irrigants reduced CFUs significantly more than saline at one, five, and ten minutes and at 200 and 400 µm depths (*p* < 0.05) (Table 1 and Table 2). PN300 was more effective in reducing CFUs compared to saline, P100, P300, and PN100 (*p* < 0.05) excluding 6% NaOCl and 2% CHX (*p* > 0.05) at all time intervals and both depths. At five minutes, 6% NaOCl and 2% CHX were the most effective among all groups (*p* < 0.05); however, no significant difference was found between PN300, 6% NaOCl, and 2% CHX at 10 min (*p* > 0.05). PN100 was more effective compared to saline and P100 at all time intervals and both depths. However, there was no significant difference observed between PN100 and P300 at one minute and five minutes at 200 and 400 µm depths. At ten minutes, PN100 showed more effectiveness than P300 at 400 µm depth. The 6% NaOCl, CHX, and PN300 were more effective than PN100 at all time intervals and both depths (p < 0.05). Mean reduction in CFUs was the highest at ten minutes followed by five minutes and minimum at one minute in all groups except saline at 200 µm (Figure 2A) and 400 µm dentinal tubule depth (Figure 2B).

**Figure 3:**
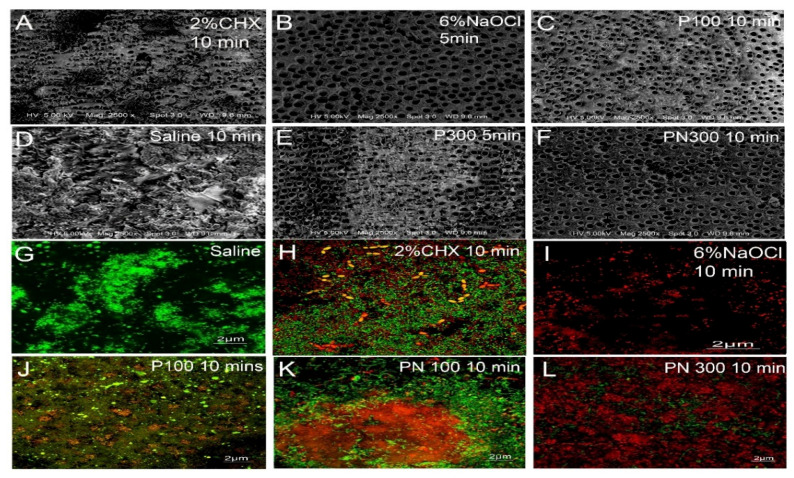
Representative images of (**A**) 2% CHX, (**B**) 6% NaOCl, (**C**) P100, (**D**) saline, (E) P300, and (**F**) PN300 treated on dentin samples. Confocal images of **(G)** saline, (**H)** 2%CHX, (**I**) 6%NaOCl, (**J**) P100, (**K**) PN100, and (**L**) PN300 showing variations in effect on biofilm grown.

SEM images verified the presence of a thick biofilm of bacteria when treated with saline. At five and ten minutes, the saline group showed the highest *E. faecalis* coverage of 67–100% on SEM images of the canal wall. At five minutes, 6% NaOCl and 2% CHX showed the least *E. faecalis* coverage of 5–33%, while PN300 showed 34–66%. At ten minutes, PN300, PN100, 6% NaOCl, and 2% CHX showed the least *E. faecalis* coverage of less than 5% (Figure 3). CLSM images showed the highest number of dead cells in dentin with PN300 compared to PN100 and saline (no dead cells) after ten minutes of exposure (Figure 3). The number of live bacteria in biofilms decreased significantly with PN300 (*p* < 0.05) and the highest dead cell count was observed in the PN300 groups (Table 3).

The antibacterial effect of PN as an endodontic irrigant in clinical samples was assessed and the reduction in CFUs count was significant in all groups compared to the control group. At one minute, 6% NaOCl (2.5 × 10^3^) showed the lowest CFUs of *E. faecalis* followed by 2% CHX (3.0 × 10^3^), PN300 (3.2 × 10^3^), PN100 (7.2 × 10^3^), P300 (8.0 × 10^3^), P100 (9.4 × 10^3^), and saline (3.0 × 10^6^). At five and ten minutes, PN300, PN100 6% NaOCl, 2% CHX, P100, and P300 showed no growth of *E. faecalis* when compared to saline (2.3 × 10^6^) (Figure 2C).

**Figure 4:**
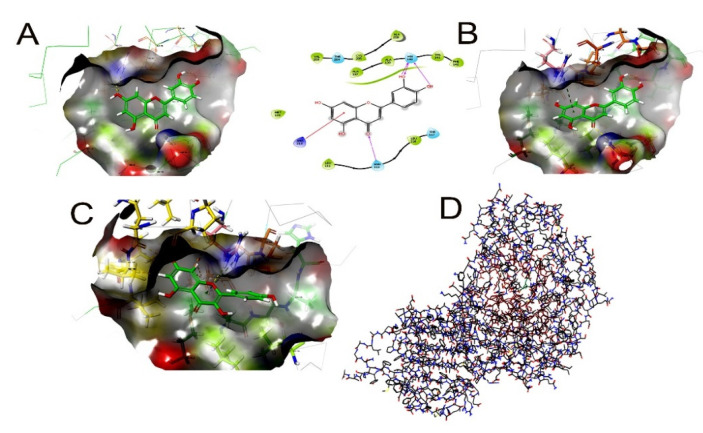
Molecular docking simulation on propolis compounds in Sortase A (4TQX) and -galactosidase (6TBI) (**A**,**B**) 4TQX SP3D HSA with propolis interaction with Sortase A. (**B**) Pinocembrin has a binding energy of 10.14 and IFD score of −445.17. The secondary structure is shown with their sub-domains identified. (**C**) The interaction of Kaempferol with neighboring residues in sub-domain 6TBI extracted from the docking results with values for HSA (black color) and b-galactosidase complex (red color) during 30 ns MD simulation (**D**) The interaction of Quercetin_ 6TBI _XP_3D.

Detailed observations of the docking poses of the bioactive compounds and their interactions with key residues of the binding site in all the three docking protocols (SP, XP, and Induced-Fit) revealed that the interactions were consistent with reasonable docking and IFD docking scores (Table 4). The negative scores indicate that the binding of bioactive compounds within sortase A and b-lactamase is favorable (Table 4). The 2D and 3D interaction diagrams of the bioactive compounds within the active site, the docking scores, IFD scores, the receptor residues involved in binding, and the nature of the interaction (Hydrogen bonding, hydrophobic bonding, etc.) are shown in Figure 4. The molecular docking results are highly favorable to postulate that the bioactive compounds form stable and strong interactions with amino acid residues in the binding sites of MMP-2 and MMP-9.

## 4. Discussion

The eradication of bacteria by endodontic treatment has been difficult primarily due to the complex root canal system and biofilm formation [2]. The success of endodontic treatment depends on chemomechanical disinfection which eliminates vital or necrotic pulp tissue, killing microorganisms in the root canal system and disrupting the microbial biofilm, leading to elimination of the etiological factors responsible for endodontic infections. Therefore, root canal instrumentation is always accompanied with copious irrigation to achieve chemical, mechanical, and biological effects [37]. In this study, all endodontic irrigants killed significantly more bacteria than the saline used as a negative control. PN300 was more effective compared to all other groups except 6% NaOCl and 2% CHX at all time intervals and both depths. This could be due to the propolis nanoparticles’ sizes, large surface-area-to-mass ratio, and very good reactivity leading to better penetration into the dentinal tubules as compared to propolis alone [25,38]. Ethanol extracts of propolis have shown a high antibacterial property mainly due to the presence of flavonoids such as pinocembrin, quercetin, and galangin that probably act on the microbial membrane or cell wall site, causing functional and structural damages [20,22,39]. Results from CLSM and CFU have shown that the propolis nanoformulation (PN300) has a significant antimicrobial effect against the *E. faecalis* biofilms, which is one of the commonly isolated bacteria associated with endodontic infections [40]. The CLSM analysis suggested green and red fluorescence intensities within the endodontic biofilms. The red fluorescence is primarily due to SYTO9 emission bands within the red wave region [41]. Most of the bacteria were alive in the control saline groups whilst the majority of bacteria showed fluoresced red in the experimental irrigant groups, more specifically in the PN300 groups. Hence, the null hypothesis that propolis had no antimicrobial effect on biofilm species was rejected.

Previous studies [42,43,44,45] have also observed an antibacterial property of propolis similar to sodium hypochlorite and CHX. At five minutes, 6% NaOCl and 2% CHX were the best among all groups; however, no significant difference was found between PN300, 6% NaOCl, and 2% CHX at ten minutes. These results are in accordance with previous studies showing 6% NaOCl to be more effective against *E. faecalis* and disrupting biofilm [46,47,48]. Similarly, 2% CHX has also been found to be effective against *E. faecalis* [49,50,51,52]. Chlorhexidine digluconate is a bisphenol compound that has a lower grade of toxicity compared to sodium hypochlorite and sustained action [47]. Although 2% CHX has been proven to promote higher antimicrobial effectiveness by reducing the bacterial load when compared to 6% NaOCl, it is unable to disrupt biofilms [52]. Moreover, CHX is water soluble and can leach out from the bonded interfaces resulting in aged loss of antimicrobial effectiveness, and even protease inhibitory activities. The gluconate salt form of CHX, which is a biguanide compound, is a strong base and poorly soluble [53] as suggested earlier. Additionally, NaOCl is a known irritant to periapical tissues and has shown re-growth of *E. faecalis* bacterial biofilms after its use [54]. That being said, *E. faecalis* infections survive harsh environments with reinfection prevailing up to 24–77% [55].

PN100 was more effective compared to saline and P100 at all time intervals. However, 6% NaOCl, CHX, and PN300 were significantly better than PN100. This can be attributed to the higher concentration of propolis used in PN300. Propolis 100 was less effective than PN100 and PN300 at all time intervals and both depths. The authors speculate that this could be due to the poor penetrability of propolis in the dentinal tubule compared with propolis nanoparticles. The higher concentration of propolis, P300, could show a similar antibacterial effect as PN100 except at ten minutes at 400 µm depth.

The signature Raman signals, first reported by Daood et al. 2016 [56] in dental biofilms, at 484 cm^−1^, were assigned to the polysaccharides seen in all samples. Based on the comparison seen amongst different specimens, the PN300 groups showed the least intensity. These signature peaks disappeared amongst the PN300 samples, endorsed by the percentages of live and dead bacteria as shown in Table 3. The obtained spectrum has multidimensional information, hypothesizing that more bacterial colonies are being affected at higher concentrations of PN100 and PN300 (*p* < 0.05). The concentrations chosen for this research such as PN100 and PN300 correspond to the minimum inhibitory concentrations and minimum bactericidal concentrations of propolis used in other similar studies [57,58]. Additionally, Bueno-Silva et al. [59] found the minimal inhibitory concentrations of propolis to vary from 15.6 to 125 μg/mL; bactericidal concentrations varied from 31.2 to 500 μg/mL. Furthermore, the concentration of propolis influences the effectiveness of PN in reducing *E faecalis* as shown in the CFUs results. Besides factors such as strength, time, and frequency, the factor volume of an endodontic irrigant influences its antibacterial effect as demonstrated by Gazzaneo et al. [60] where high volume of an irrigant had a positive influence on intracanal disinfection. Therefore, to standardize the volume factor throughout the investigation, 5 mL of each endodontic irrigant was used. The antibacterial effect of PN against *E. faecalis* isolates from patients with failed root canal treatment also showed good results mainly due to the direct exposure of irrigants instead of using the tooth model. Haapasalo et al. [61] demonstrated the effect of dentin on the antimicrobial properties of endodontic medicaments. They concluded that most of the disinfecting agents can rapidly kill the microbes when tested in vitro in a test tube, but the effectiveness of the same agents can be weaker in in vivo conditions. This is mainly due to the interaction of endodontic disinfecting agents with dentin and other compounds such as serum proteins, hydroxyapatite, collagen, and microbial biomass.

In this study, the extracted bovine tooth model developed by Haapasalo and Ørstavik was modified to include natural human teeth as specimens. This provided a better simulation of clinical settings to assess the antibacterial effectiveness of irrigants in the dentinal tubules [13]. Additionally, the samples were tested at two depths of dentinal tubules—200 and 400 µm—because previous studies have shown that dentin has an inhibitory effect on the antibacterial effectiveness of endodontic irrigants [62,63,64]. Therefore, the survival of the bacteria could be attributed to their invasion into the varying depths of dentinal tubules against irrigants [65,66]. The present study determined the effective duration of various irrigants tested at three different time intervals because of their time-dependent antimicrobial effect [67,68]. This characteristic could be useful in clinical practice to efficiently disinfect the root canal system. *E. faecalis* was chosen as it has been one of the most prevalent (24% to 77%) microorganisms isolated from failed root canal cases [66,69,70]. In this study, the effectiveness of endodontic irrigants was assessed against a 21-day mature biofilm because it has been shown that mature *E. faecalis* biofilms in dentin canals at 21 days are more resistant to endodontic irrigants than young biofilms [8]. The mechanism of resistance of the older biofilm is complex and may involve various mechanisms [70]. In the present study, an *E. faecalis* mono-species biofilm has been used which is in accordance with Swimberghe et al. [71] who presented an outline of laboratory root canal biofilm model systems and critically appraised the factors that constitute these models. The authors found that the ability to reduce intracanal bacteria is ascribed to this new strategy indicative of a favorable endodontic regimen, showcasing new versatile PN300-based materials and a potential use in endodontics. Studies on the effectiveness of PN300 as an irrigant are underway against polymicrobial biofilm disruption with future animal studies.

### Limitations and Future Directions

In this study, the antimicrobial effectiveness of PN300 as an irrigant has been evaluated against a single-species biofilm. Although *E. faecalis* can be found in cases of persistent endodontic infections, typically, most endodontic infections are multi-species biofilms. Therefore, future studies should be evaluated against a polymicrobial biofilm and its disruption. To further strengthen the evidence, future animal studies and clinical trials are warranted.

In this study, the antibacterial effect of PN as an endodontic irrigant was evaluated against *E. faecalis* isolates from patients with failed root canal treatment. This is a first of its kind project in which the effectiveness of PN against *E. faecalis* isolates from patients with failed root canal treatment was evaluated. Therefore, the first step to assess their effectiveness on planktonic bacteria was carried out in this study as a preliminary screening of these new disinfectants before proceeding onto more complex biofilm designs. However, further experiments in the future can be carried out to evaluate the effectiveness of PN against a polymicrobial biofilm using extracted teeth or even in animal model.

## 5. Conclusions

PN300 as an endodontic irrigant was equally as effective as 6% NaOCl and 2% CHX in reducing *E. faecalis* CFUs in a human tooth model and *E. faecalis* isolates obtained from patients with failed root canal treatment. Therefore, PN300 can be proposed as an alternate endodontic irrigant.

PN100 as an endodontic irrigant was more effective in reducing *E. faecalis* CFUs than saline and P100 at all time intervals and both depths. PN100 was equally as effective as P300 at one minute and five minutes and less effective than 6% NaOCl and 2% CHX at all time intervals and depths.

PN300 and PN100 as endodontic irrigants were the most effective at ten minutes in reducing *E. faecalis* CFUs when compared to one minute and five minutes.

## Figures and Tables

**Table 1:** Comparison of mean rank in between groups at 1, 5, and 10 min, 200 µm (Kruskal–Wallis).

Group	Saline	P100	P300	PN100	PN300	6% NaOCl	2% CHX	*p* Value
1 min	65.50	53.00	40.60	33.00	24.00	15.80	16.60	<0.001
5 min	65.50	49.25	41.10	35.55	30.30	12.95	13.85	<0.001
10 min	65.50	55.30	43.00	33.10	19.85	16.30	15.45	<0.001

**Table 2:** Comparison of mean rank in between groups at 1, 5, and 10 min, 400 µm (Kruskal–Wallis).

Group	Saline	P100	P300	PN100	PN300	6% NaOCl	2% CHX	*p* Value
1 min	65.50	52.75	39.00	37.10	23.55	15.90	14.70	<0.001
5 min	65.50	53.35	43.95	36.00	22.80	15.15	11.75	<0.001
10 min	65.50	54.40	46.60	30.70	20.65	15.85	14.80	<0.001

**Table 3:** Bacterial viability in single-species *E. faecalis* biofilms following different disinfectant treatments.

Group	Dead *E. faecalis*			Live *E. faecalis*		
	Mean %		SD	Mean %		SD
Saline	0.89	A	0.33	99.11	I	12.3
2% CHX	53.4	B	8.1	46.6	II	6.9
Propolis 100µg/mL (P100)	60.7	C	6.89	39.3	III	8.7
Propolis 300µg/mL (P300)	65.4	C	7.1	34.6	III	6.6
Propolis Nanoparticle 100µg/mL (PN100)	86.7	D	11.33	13.3	IV	3.3
Propolis Nanoparticles 300µg/mL (PN300)	93.2	E	9.1	6.8	V	2.1
6% Sodium Hypochlorite (6% NaOCl)	79.7	F	7.8	20.3	VI	6.5

CHX—chlorhexidine; P—propolis; PN—propolis nanoparticle. Values are means ± standard deviation. Groups identified by different numerals and letters were significantly different at *p* < 0.05.

**Table 4:** Identification of key binding poses of the Sortase A and β-galactosidase active sites involved in binding with propolis. Sequences established as per number of residues. Synthetic and protein ligands were removed, as well as crystallographic water molecules.

	Sortase A (PDB ID: 4TQX)				β-Galactosidase (PDB ID: 6TBI)			
	SP Score	XP Score	IFD		SP Score	XP Score	IFD Score	
			XP Score	IFD Score			XP Score	IFD Score
Pinocembrin	−5.978	−4.836	−10.149	−445.17	−5.424	−3.492	-	-
Kaempferol	−6.104	−5.307	−7.423	−442.38	−6.247	−5.984	-	-
Quercetin	−5.761	−6.67	−7.851	−441.54	−5.393	−4.924	-	-

## Data Availability

The data analyzed during this present study are available from corresponding author on request.

## Abbreviations

CFU	Colony Forming Units
CHX	Chlorhexidine
CLSM	Confocal Laser Scanning Microscopy
*E. faecalis*	*Enterococcus faecalis*
EDTA	Ethylene Diamine Tetra Acetic acid
MTAD	Mixture of Tetracycline, Acid and Detergent
NaOCl	Sodium Hypochlorite
P100	Propolis 100 µg/mL
P300	Propolis 300 µg/mL
PN100	Propolis Nanoparticle 100 µg/mL
PN300	Propolis Nanoparticle 300 µg/mL
SEM	Scanning Electron Microscope
*w*/*v*	weight per volume
TSB	Tryptic Soy Broth
kV	kilo Volt
*v*/*v*	volume per volume
